# FlaGs and webFlaGs: discovering novel biology through the analysis of gene neighbourhood conservation

**DOI:** 10.1093/bioinformatics/btaa788

**Published:** 2020-12-12

**Authors:** Chayan Kumar Saha, Rodrigo Sanches Pires, Harald Brolin, Maxence Delannoy, Gemma Catherine Atkinson

**Affiliations:** Department of Molecular Biology and Umeå Centre for Microbial Research, Umeå University, Umeå 901 87, Sweden; Department of Chemistry, KTH Royal Institute of Technology, Stockholm 100 44, Sweden; Department of Molecular and Clinical Medicine, Wallenberg Laboratory, University of Gothenburg, Gothenburg 413 45, Sweden; Département Génie Biologique, Campus SophiaTech, Université Nice Sophia Antipolis, Nice 06900, France; Department of Molecular Biology and Umeå Centre for Microbial Research, Umeå University, Umeå 901 87, Sweden

## Abstract

**Summary:**

Analysis of conservation of gene neighbourhoods over different evolutionary levels is important for understanding operon and gene cluster evolution, and predicting functional associations. Our tool FlaGs (standing for Flanking Genes) takes a list of NCBI protein accessions as input, clusters neighbourhood-encoded proteins into homologous groups using sensitive sequence searching, and outputs a graphical visualization of the gene neighbourhood and its conservation, along with a phylogenetic tree annotated with flanking gene conservation. FlaGs has demonstrated utility for molecular evolutionary analysis, having uncovered a new toxin–antitoxin system in prokaryotes and bacteriophages. The web tool version of FlaGs (webFlaGs) can optionally include a BLASTP search against a reduced RefSeq database to generate an input accession list and analyse neighbourhood conservation within the same run.

**Availability and implementation:**

FlaGs can be downloaded from https://github.com/GCA-VH-lab/FlaGs or run online at http://www.webflags.se/.

**Supplementary information:**

Supplementary data are available at *Bioinformatics* online.

## 1 Introduction

Conservation of gene order at long evolutionary distances is a strong indicator of a functional relationship among genes (Overbeek *et al.*, 1999). Extreme examples are the tryptophan biosynthesis (Dandekar, 1998), and *str* ribosomal protein operons (Lechner *et al.*, 1989), which are conserved from bacteria to archaea. The vast amount of genomic sequence data that has become available in recent decades is a treasure trove of clues about the function of uncharacterized proteins, and the pathways in which they are involved (Gabaldon and Huynen, 2004). High-throughput identification of gene order conservation in genomes is a promising approach for predicting the involvement of proteins in particular pathways or systems. In addition to yielding functional predictions, the identification of conserved genomic architectures is essential for understanding the evolutionary dynamics behind the formation and restructuring of gene clusters, including reassembly of operons after disruption during evolution (Omelchenko *et al.*, 2003).

While there are a range of tools that analyse gene neighbourhood conservation or integrate this data along with other metrics for functional association prediction, these tend to be either restrictive in the genomes that can be considered (e.g. only complete genomes or those of model organisms) or require the creation of local genome databases (Garcia *et al.*, 2019; Lemoine *et al.*, 2008; Martinez-Guerrero *et al.*, 2008; Overmars *et al.*, 2013; Szklarczyk *et al.*, 2015). Other tools that connect to the National Center for Biotechnology Information (NCBI; https://www.ncbi.nlm.nih.gov/) to detect operons may lack sensitive sequence searching for homology assignments of neighbourhood genes (Gumerov and Zhulin, 2020). We felt there was a need for a tool that allows the use of the huge quantity of publicly accessible data in the NCBI RefSeq database (O'Leary *et al.*, 2016) and is sensitive enough to answer questions about homologous proteins over any evolutionary distance, from the strain or isolate level, to inter-kingdom or even inter-domain comparisons. We set out to build a Python tool that fulfils our list of essential criteria:

1. allows the user to have complete control over the input genomes being analysed;
2. has a simple input format that does not require coding, downloading of genomes or formatting of databases;
3. nevertheless, also has the option of running using locally stored genomes for offline analyses or analysing genomes that are not public;
4. can be run via a server with results emailed to the user;
5. can detect remote homology, suitable for analysing the most distant relationships among proteins and taxa as well as closer comparative analyses;
6. outputs gene neighbourhood annotated onto a phylogenetic tree;
7. produces publication-quality editable vector graphics.

## 2 The FlaGs workflow

**Fig. 1.**
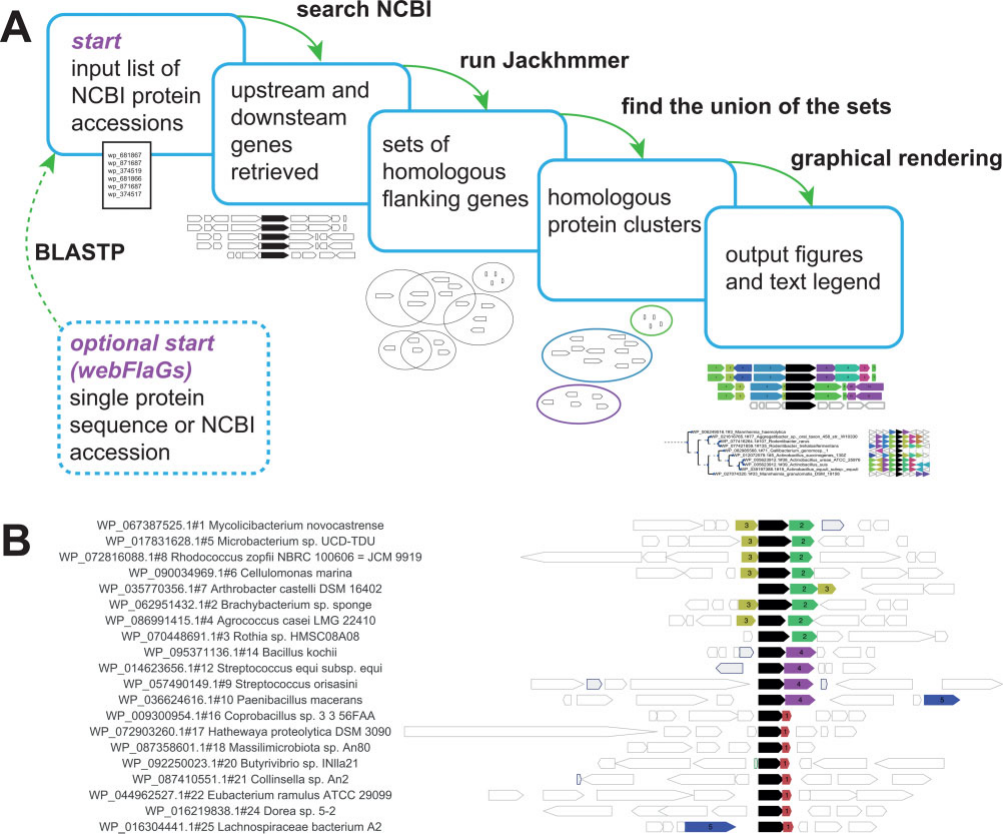
The FlaGs workflow and example results. (**A**) The user inputs a list of protein accession numbers—optionally with GCF assembly IDs—and can specify the number of adjacent flanking genes to consider, and the sensitivity of the Jackhmmer search through changing the E value cut-off and number of iterations. The web version of FlaGs (webFlaGs) can optionally use a single protein sequence or NCBI accession and begin by executing a BLASTP search against the RefSeq database (excluding eukaryotes) or a representative genome database to generate the input list of accessions. The output always includes a to-scale figure of flanking genes, a description of the flanking gene identities as a legend, and optionally, a phylogenetic tree annotated with colour- and number-coded pennant flags. (**B**) Example results using toxins of the toxSAS toxin–antitoxin system (Jimmy *et al.*, 2020) as the query. Empty genes with grey borders are not conserved in the dataset, and grey genes with blue borders are pseudogenes. In this example, FlaGs reveals four different homologous groups of antitoxins as flanking genes, two of which (green and yellow) are antitoxins for the same cognate toxin. Group number 5 is an integrase. As FlaGs does not require complete genomes, regions can lack flanking genes on one side if the query gene is close to the end of a contig, as is the case with *Arthrobacter castelli* in this example

Our resulting tool that fulfils the above requirements is called FlaGs (standing for Flanking Genes) (Fig. 1A). FlaGs takes in user-determined NCBI accessions that link to the RefSeq database (around 170 million proteins from almost 100 000 organisms as of March 2020). Input files can be easily and quickly prepared from selected sequences in the output of an NCBI BLASTP search against the RefSeq database without any scripting (see the manual; Supplementary Materials File S1). An optional addition to the input file is the NCBI genome assembly identifier to target a particular genome. FlaGs clusters flanking gene-encoded proteins using the sensitive Hidden Markov Model-based method Jackhmmer, part of the HMMER distribution (Eddy, 2011). There are three ways to run FlaGs:

1. through the web server at www.webflags.se. This method can optionally include a BLASTP search against microbial RefSeq genomes or a representative genome database to identify homologues which with to run FlaGs (Camacho *et al.*, 2009);
2. locally, with FlaGs querying NCBI as it runs, and not requiring locally stored genomes;
3. locally, using locally stored genomes in RefSeq GFF and protein FASTA format.

FlaGs outputs information on the conservation of flanking gene-encoded proteins, and their identity, in graphical and text formats (Fig. 1A). The output always includes a to-scale diagram of flanking genes, number- and colour-coded by conservation groups (Fig. 1B). A ‘description’ file is also included, which acts as a legend for interpreting the flanking gene diagram. An optional output is a phylogenetic tree annotated with flanking genes reduced to triangular pennant-like flags. The tree-building feature uses the ETE 3 Python environment (Huerta-Cepas *et al.*, 2016).

FlaGs is a flexible tool for sensitive detection of flanking gene conservation at any evolutionary distance, and displays results in an intuitive, publication-quality vector graphics format. The utility of FlaGs is exemplified by our recent discovery of a novel toxin–antitoxin system exploiting growth control via ppApp alarmone nucleotide signalling (Jimmy *et al.*, 2020). The web server STRING is one of the most widely used tools to study the gene neighbourhood conservation of a gene of interest (Szklarczyk *et al.*, 2015). STRING’s great strength is that it brings together pre-computed association data from a number of different sources to predict functional associations. It is an excellent first port of call for predicting the function of conserved genes. STRING, however, uses a limited set of around 5000 input organisms, and does not include bacteriophages. Therefore, it is somewhat limited when addressing neighbourhood conservation of genes with extremely patchy distributions as is often the case with genes belonging to the accessory component of pangenomes. The discovery of toxSASs was only possible through the access of FlaGs to the extensive cellular and viral genome resources in the RefSeq database. We expect that FlaGs will continue to be successful in the prediction and evolutionary analysis of genomic loci with various functions, not just toxin–antitoxins, but for example, secretion systems (where it has already been used in the description of a novel system (Palmer *et al.*, 2020)), antibiotic biogenesis clusters, viral defence mechanisms, gene transfer agents, pathogenicity islands and transposons. A future direction of FlaGs is to go beyond RefSeq, taking advantage of all the genomic data stored in Genbank, which will further increase the genomes accessible to neighbourhood analysis by FlaGs by hundreds of thousands.

## Supplementary Material

btaa788_Supplementary_Data
